# S227. A PROPOSED ALTERNATIVE BETWEEN DISCONTINUATION AND MAINTENANCE OF ANTIPSYCHOTICS: A GUIDED DOSE REDUCTION TRIAL FOR PATIENTS WITH REMITTED PSYCHOSIS

**DOI:** 10.1093/schbul/sby018.1014

**Published:** 2018-04-01

**Authors:** Chen-Chung Liu

**Affiliations:** National Taiwan University Hospital and College of Medicine, National Taiwan University

## Abstract

**Background:**

Early intervention at the beginning of schizophrenia and related psychotic disorders can get better treatment response. Once symptoms subsided, the majority of patients wish to discontinue medications, yet currently the mainstream opinions still recommend maintenance antipsychotic therapy because non-adherence to medication is the most significant risk factor to predict a relapse. However, recent longitudinal studies assessing patients in community for a longer term found that discontinuation of antipsychotics might not necessarily be parallel to poorer functioning. Also there are studies suggesting a lower percentage of dopamine occupancy by antipsychotic is acceptable in stable patients with psychosis. To elucidate such discrepancies, a hypothetical compromised approach “guided dose reduction, but not aiming at discontinuation” was proposed and an observational clinical trial was initiated since July 2017.

**Methods:**

Outpatients with schizophrenia-related psychotic disorders under remitted states will be recruited and randomized into guided dose reduction group (GDR, target n = 80) and maintenance treatment group (MTG1, target n = 40), and those who eligible to dose reduction yet willing to continue medication will serve as a naturalistic observation group (MTG2, target n = 40). Patients in the GDR will reduce no more than 25% of their current dose of antipsychotics and closely monitored every 4 weeks for at least 24 weeks before next dose reduction adjustment. Patients of both MTGs receive treatment as usual. All patients will be followed up for at least 2 years. The main outcomes of interests are differences in relapse rates, personal social performance, quality of life, drug-related adverse reactions, medication satisfaction, and neurocognitive functioning between groups. Patient’s actual medication status will be monitored by keeping a log and therapeutic drug monitoring on selective antipsychotics. Patient’s demographics and clinical variables will be taken to test whether these variables are related to outcomes during follow-up.

**Results:**

Currently 26 patients have participated in this study, including 10 males and 16 females, with a Mean (SD) age 31.8(7) years old. Eleven of them were in GDR group, 10 in MTG1, and 5 in MTG2. Their baseline PANSS scores were 36.9(5.7), 37.4(7.9), 49.2(7.4), CGI-S scores were 1.7(0.6), 1.5(0.7), 2(0), and Personal Social Performance (PSP) scores were 82.2(7.9), 83.9(6.8), 77.8(2.3) in GDR, MTG1, and MTG2, respectively. So far one patient in the GDR group has resumed her original dose due to suspected early signs of relapse and no further worsening of symptoms noticed, while one patient of the MTG1 withdrew consent due to feeling unnecessary to receive comprehensive follow-up assessments. Most of patients endorsed no significant difference between ordinary dose and reduced dose at present time.

**Discussion:**

During the first 4 months of this trial, we have not seen any unexpected happening yet. We will continue case recruitment and follow-up to test if the metaphor derived from Cantor’s Set and Sierpinski Triangle can serve a valid model for our dose reduction trial and see if such a slow-paced guided dose reduction approach a feasible solution for the debates between medication discontinuation and maintenance.

